# Georgia State Medical Association

**Published:** 1892-05

**Authors:** 


Secretary.

1603 N. Broadway, Baltimore, Md.

### GEORGIA STATE MEDICAL ASSOCIATION.

FORTY-THIRD ANNUAL MEETING, HELD AT COLUMBUS, APRIL 20, 21 AND 22, 1892.

### FIRST DAY—MORNING SESSION.

The Association convened in Springer's Opera House, and was called to order at 11:25 A. M. by the President, Dr. G. W. Mulligan, of Washington.

THE ADDRESS OF WELCOME was delivered by the Hon. J. J. Slade, Mayor of Columbus, which was responded to by Dr. J. B. Baird, of Atlanta.

Prayer was then offered by the Rev. Robert Harris.

President Mulligan delivered his annual address, in which he pleaded for the abandonment of all unscientific methods of reasoning and investigation, and for a still firmer alliance with those principles which, in other branches of knowledge, have produced such brilliant successes. He pleaded for a less reliance on the blind gropings of empiricism and a still closer affiliation with every method of modern science. He also pleaded for the exclusion of all fallacies and the adoption of every safeguard which may indicate a wrong direction in our studies, and for the entire displacement of self in the investigation of life, both in health and disease, and a concentration of all our powers in searching out and fortifying the laws governing the relation and succession of these phenomena.

DR. J. M. SPENCE, of Waresboro, read a short paper entitled "A Case of Ovarian Cyst."

May 1, 1890, he was called in consultation with Dr. W. P. Williams, of Waycross, to see a case. Five years prior to his visit the patient gave birth to twins, attended, however, with great difficulty. An undergraduate attended her. Her health was apparently good until she became pregnant again twelve months hence. The issue of the second pregnancy was triplets, the patient again enduring a state of prolonged labor and excruciating pain under the supervision of the same quack. In about two months something began to grow in the region of the right ovary. It grew rapidly for about three years. After a careful examination, an exploring needle was introduced, which, on its withdrawal, was found to contain a thick, dark straw-colored fluid, about the consistency of ordinary syrup. The legs and whole lower extremities were infiltrated with water, her tongue coated, and bowels costive, the infiltration being caused by compression of the tumor on the kidneys and ureter. They next took a measurement of the abdomen, which was six and one-half feet, and from the pubes to the ensiform appendix the distance was three feet and nine inches. Calomel, pulverized rhei, carbonate of soda in repeated doses, together with iron, strychnine and digitalis, were given for a few days. Six days later they met and introduced a trocar and canula through the walls of the abdomen, and through the aperture thus made there came seventy quarts (which seems almost incredible) of a thick straw-colored fluid. The same treatment was continued at their second visit, two weeks later, and at this time twenty-eight quarts of a similar fluid were withdrawn, together with something that looked like human brains. At a third visit, two weeks later, they drew thirty quarts of similar fluid, but the trocar would frequently become choked with the brain-looking matter discovered after the operation. Ten days later the patient was seized with cholera morbus and died.

DR. D. C. HURT, of Columbus, followed with a paper on "The Influence of Civilization and Social Life upon Women as Viewed from a Medical Standpoint." (Will appear next month.)

DR. H. PERDUE, of Barnesville, read a paper on "The Treatment of Pneumonia with Report of Cases."

Since the first of January, he had had, besides several of the lobular variety, fifteen cases of the lobar. Three of the lobar class had one lobe of the lungs affected, four had two, seven had three, and one had four. This shows an unusual *pro rata* number of lobes attacked, yet he had not lost a single case.

In lobar pneumonia he uses peroxide of hydrogen with good effect. It is given for the germicidal properties, the oxygen it furnished the patients exhilerated and strengthened them. If it nauseates—and sometimes it does—the quantity should be diminished. He feels confident it has saved valuable lives. Throughout the stages of congestion and consolidation he gives phenacetine in five grain doses in connection with whiskey or milk punch every four hours, unless the temperature should get below 103° F. Good nursing is an important factor in the management of pneumonia patients. A nutritious liquid diet, such as sweet milk, beef tea and palatable broths, should be given. In convalescence stimulating expectorants and tonics are indicated.

In catarrhal pneumonia, the supporting treatment should be given from the beginning. He generally gives carbonate or muriate of ammonia during the entire treatment. Quinine he considers a valuable remedy in reducing temperature, and as an aid to resolution. Mild mustard poultices or blisters to the chest are valuable; also flannel cloths or jackets saturated with turpentine, diluted with oil, if the patient is a child. Great care should be exercised in convalescence to prevent a relapse or a second attack.

DR. R. R. KIME, of Atlanta, read a paper entitled "What is Gynæcology?" He briefly reviewed the progress made in gynæcology, and said the Father of Medicine recognized the sympathy existing between the mammary gland and the uterus, which he utilized by cupping them for uterine hæmorrhage.

Another twelve centuries brought us to the nineteenth century in which fads, fashions and cycles held sway as in the olden times. To illustrate: We have only to mention that a few years since everybody slit or divided the cervix; a few years later everybody sewed it up, and now everybody divulses or dilates, and drains.

A few years since everybody probed or sounded the uterus, applied caustics to ulcers of the os. Now the sound is not often used and ulcers of the os are a nonentity. Within the last three or four decades the advancement in gynæcology has been so rapid that it is difficult to keep in the line of march as the procession advances to new thoughts and new discoveries. Ovariotomy is about the only operation that has not been greatly abused or oft-resorted to without just provocation. It first had met with violent opposition, but to-day stands as an established procedure. The improved technique with lessened mortality are about the only improvements since the operation was first performed. The speaker was glad to see gynæcology as fast approaching a scientific basis. In the last two decades, it has outstripped all other specialties of medicine in new discoveries and improved operative technique, thereby saving many of "God's noblest gift, the woman perfected."

He concluded by saying that the South had not been asleep, that she furnishes and has furnished her quota of active original workers in this specialty, that the Southern Surgical and Gynæcological Association is maintained within her borders, and among her sons may be found such names as McDowell, Sims, Talliferro, and Battey, with many other eminent active workers in that line.

Adjourned.

APRIL 21.—MORNING SESSION—SECOND DAY.

DR. A. G. BLAIN, of Macon, read a paper on "Remittent Fever."

In persons exposed to the malarial miasm, it is a good plan to give daily doses of quinine (5 to 10 grains) each morning, and they should avoid night exposure. Should the practitioner see the patient in the prodromic stage of lassitude—furred tongue, foul breath—he believes that he can often prevent an attack of remittent fever by giving a mercurial cathartic, followed by quinine and a mineral acid.

ACTUAL TREATMENT. The patient should be put to bed in a well ventilated room. Patients require good nursing. Regulate diet—a fluid diet is necessary and milk is the best to rely upon. The practitioner may have to boil the milk and give it iced, or he may peptonize it when necessary. He believes it best to give all nourishment in small quantities and at frequent intervals. In excessive temperature he relies upon phenacetine as the best drug at his command, in doses of from 5 to 10 grains, as the case may demand. The pain in the limbs during convalescence can be controlled by phenacetine and salol. Convalescence will be greatly assisted by a tonic of nux vomica combined with nitro-muriatic acid and some one of the bitters.

DR. W. E. B. DAVIS, of Rome, read a paper entitled "The Surgical Treatment of Puerperal Peritonitis."

He said that puerperal fever was no longer regarded as a distinct affection, a disease *sui generis*, but is looked upon as a septic fever resulting from infection during the puerperal state. When quarts and gallons of pus are reported as having been removed from the general peritoneal cavity and recovery followed, he believes that the pus has usually resulted from a local collection, which has ruptured into the general cavity and the operation has been done before sufficient time has elapsed for this amount of pus to result from the septic inflammatory process in the general cavity. It is easy to understand how a gallon of pus, which has been shut off from the general cavity by inflammatory exudations and adhesions, and which has only recently ruptured into the peritoneal cavity, can be removed and recovery follow, and it is not difficult, Dr. Davis thinks, to comprehend how this condition might be mistaken for acute general suppurative peritonitis with a gallon or quart of pus as a result in the cavity, as the pus, by its irritating properties, will produce an inflammation which would be misleading; but the condition is quite different from what would be had, if the pus had been the result of a general inflammation. Dr. Davis maintains that localized puerperal peritonitis with pus formation is a form of the disease that is amenable to surgical treatment. He had operated on cases where there seemed to be no hope of recovery, with favorable results. He had seen cases, where the pulse was 135, get well after an operation for the relief of this condition.

Frequently the fever from puerperal infection may continue for weeks where there is no pus formation, and in which the ovaries are greatly enlarged, and the broad ligament thickened with occlusion of the tubes. He had operated on such a case where the tissues were so soft that his ligatures would cut through, and it was necessary to take up the vessels and ligate them separately. The ovary in this condition is as septic as if it contained pus. Frequently the masses felt in the pelvis following delivery or abortion are due to pelvic peritonitis with adhesions to the intestine and omentum, and when an operation is made to evacuate pus, we will often find in the place of pus this condition present, but where fever is kept up and the patient's condition shows sepsis, it is wiser to open the abdomen, break up the adhesions, and remove the diseased appendages, which are keeping up the infection.

Dr. K. P. MOORE, of Macon, read a paper entitled "How shall we Manage the Uterus after Abortion?" He emphasized the necessity of clearing out the cavity of the uterus in cases of abortion, and by so doing it would be the means of preventing some of the troubles with which the gynecologist has at present to deal.

DR. C. D. ROY, of Atlanta, followed with a paper on "Cough; Some of its Causes, and Treatment."

After enumerating and dwelling upon the causes of cough, he touched upon the treatment. Every source of possible irritation should be found and removed. Hypertrophies were best removed with the galvano-cautery, and polypi should be thoroughly extirpated with the wire ecraseur. All septum deviations and spurs, when prominent enough to be a source of irritation, should be removed by whatever method the operator chooses.

The speaker called attention to a cough which is sometimes present about the age of puberty, seemingly dependent upon the condition of the reproductive organs or that of the blood.

In the *Lancet* of December, 1890, Sir Andrew Clark cited several cases of this condition occurring in his practice, both in boys and girls. Dr. Leaming, of New York, who has since called attention to this subject, has reported such a condition occurring in a young girl suffering with chlorosis, following an attack of mountain fever. The late Sir Morrell Mackenzie has also reported such a case. That there is a close relationship between certain conditions of the reproductive organs and certain nervous phenomena in other portions of the body, no one will deny, for day by day the correlation of our bodily functions is growing in importance. Dr. John Mackenzie, of Baltimore, has called attention to certain nasal conditions coexisting with a peculiar state of the reproductive organs, which from their constancy do not seem to be entirely fortuitous. In all such cases the treatment must be general, the aim of the physician being to restore the body to as normal condition as possible by rectifying all morbid states in individual organs.

Adjourned.

SECOND DAY—AFTERNOON SESSION.

DR. A. W. CALHOUN, of Atlanta, read a paper entitled "Some Observations upon Cataract Operations and After Treatment."

He reported 904 operations for cataract. The records of his first 47 cases had been lost, so that the report simply embraced the records of 847 cases. Of this number 203 were cases of soft cataract in children, and 654 cases of hard senile cataract in adults. The ages of the children ranged from three months to 25 years; the ages of the adults ranged as follows: 138 from 25 to 40 years of age; 82 from 40 to 50 years of age; 98 from 50 to 60 years of age; 157 from 60 to 70 years of age; 104 from 70 to 80 years of age; 28 from 80 to 90 years of age; 11 from 90 to 94 years of age, making 654 of hard cataracts, which, including 203 soft cataracts, makes 904. In the beginning of his cataract operations, Dr. Calhoun said if he got 95 per cent. of successes he thought he was doing well; but since the injection of cocaine and the advent of antiseptic surgery a hundred per cent. of successes has been the result in his cases.

Dr. A. A. SMITH, of Hawkinsville, read a paper on "Gunshot Wound of the Stomach with Report of a Case." The patient, a negro, had received a pistol shot said to be in the stomach. After careful examination the doctor found that in the fight, which had occurred two hours previously, his patient had received a wound directly over the stomach. A casual examination with the eye alone was all that was necessary to show that the ball had penetrated the cavity of that organ. A more thorough examination was made with the probe, and it was found that the ball had entered at a point about two inches below and to the left, at the tip of the ensiform cartilage of the sternum. The probe passed readily into the cavity of the stomach, and the doctor supposed that the ball had passed through and lodged in some other portion of the body, and did not pursue the examination further. At a third visit, which was about forty-eight hours after the boy had received the shot, he ascertained that there had been two or more free evacuations from the bowels, and to his great surprise, the ball had passed with one of these evacuations. The wound healed by first intention, and just one week from the date of the injury the boy returned to his work.

Dr. R. O. COTTER, of Macon, read a paper the title of which was "Some Practical Points in Reference to Eye Strain."

He was impressed with the observation, that the vast majority of general practitioners overlook the easily substantiated fact, that such a great multitude of morbid reflex neuroses, are brought about by eye strain; that is to say, uncorrected errors of refraction. He referred especially to hyperopia and hyperopic astigmatism. Myopes generally complain of not being able to see well enough at a distance. Headache was by far the most common accompaniment of hyperopia and astigmatism. These headaches were most common in the temples, frontal region and vertex. They frequently appeared to be what are known as sick or bilious headaches, and are in any case truly distressing in character. Correcting refractive errors was the most prominent and by far, the most scientific part of an oculist's work, and he feared that many practitioners overlooked these cases; that they met with one hundred of such sufferers, where they saw one case of cataract.

DR. R. J. NUNN, of Savannah, read a paper entitled "Preliminary Observations on the Behavior of Iodine in the Presence of Camphor, Menthol, Thymol," etc.

*Alcohol.*—If we take a saturated alcholic solution of iodine, add to it as much camphor as it will dissolve, and its solvent capacity for iodine is immensely increased. In fact, it will be found that of a saturated solution of camphor in alcohol 125 minims will dissolve 50 grains of iodine.

*Carbolic acid.*—Liquify a quantity of carbolic acid with a little alcohol (about one per cent.), saturate the liquified carbolic acid with iodine, next saturate this iodo-phenic solution with camphor, of which it will dissolve 300 to 400 per cent. This camphorated iodo-phenol will now dissolve or appropriate iodine until it becomes a dense syrupy liquid; or

*Experiment 3.*—Prepare a saturated solution of camphor in carbolic acid (this formula, which is original with Dr. Nunn, and has been used by him for many years, is possessed of valuable medicinal properties, and is known locally as phenocamphique), the result of the saturation of this solution with iodine will be similar to that obtained in experiment No. 2.

*Salol.*—If camphor and salol are mixed in proper proportions a liquid results, which also has the power of dissolving iodine in large quantity, a blackish liquid resulting.

*Thymol.*—Thymol liquifies when mixed with camphor, and the resulting fluid is also a powerful solvent of iodine.

*Menthol.*—A mixture of menthol and camphor also liquifies, and in this liquid iodine is very soluble.

DR. WILLIS F. WESTMORELAND, of Atlanta, read a paper on "Plaster of Paris in Surgery."

The object of the paper was not to discuss the various appliances adopted by different surgeons, but to present what he regarded as the best plan; one that showed results in every particular more satisfactory to the surgeon and patient than any other plan that has been presented in the past or present. It was not every plaster of Paris splint (so-called) that is applied to a fractured bone that does well. It must be properly applied, of appropriate material, with the fragments of the fractured bone properly adjusted to give the results which are so much to be desired. For a time this splint was used only in simple fractures, but now surgeons use them in compound comminuted fractures with the very best results, often saving a life or a limb that was formerly sacrificed to the knife, or other plans of treatment that resulted in the death of the patient. Dr. Westmoreland then gave the details of its application.

Adjourned.

APRIL 22—THIRD DAY—MORNING SESSION.

The first paper read was by Dr. L. G. Hardman, of Harmony Grove, entitled "Eclampsia after Delivery with Report of Cases," in which he summarized as follows:

1. That eclampsia does frequently occur after delivery.

2. That judging from the cases presented it occurs for the most part in patients with pyelitis.

3. That when we find pus in the urine of pregnant women and can exclude specific disease of the kidneys, any bladder or urethral disease likely to cause it, and thus feel pretty certain that the pus comes from the kidney; and if it continues and begins to cause any general disturbance of the health, he believes it is our duty to induce abortion or premature labor, and thus save our patient a great risk.

DR. A. G. HOBBS, of Atlanta, followed with a short paper on "Some Remarks on Tonsil Excisions, with Presentation and Description of a New Instrument."

The instruments exhibited were the practical outcome of the speaker's efforts through several years to do away with some of the objections to the instruments most commonly used for excising hypertrophied tonsils, as the ring tonsillotomes, the guillotines, wire loops, etc. He still, however, uses the ring blades in young children and in other cases where the tonsil projects decidedly with a base but little, if at all, larger than the body.

The objection to the guillotine or ginged instruments, was that the latter, especially, so often leave a margin of bruised tissue, which in many cases ends in slough; and, with neither of them is the operator quite certain of the exact amount of the tonsil he will clip, especially if the attempt is made on a mass that is soft, broad and nodulated. The cold wire snare causes great pain and requires a much longer time, and, moreover, it leaves a contused stump unless the whole organ be extirpated, which he never does intentionally. The hot wire, even with the most approved guards, endangers the pillars and is, like the cold wire, quite a formidable instrument, at least, in the mind of the patient.

The blades of the instrument exhibited were curved both on the flat and on the edge and are in pairs, right and left. Either blade may, however, be used on either tonsil, especially if the operator is ambidextrous, by making the section upward or downward according to the curve. But in most cases it is easiest and safest to make the section upward to avoid cutting the base of the tongue; the flat curve of the blade will prevent the danger to the posterior pillar. The firm hold by the double hook enables the operator to lift the mass of tonsil tissue out of its bed from between the pillars to any extent, even when it is partly overlapped by large pillars. In a total of something over four thousand tonsil excisions, Dr. Hobbs finds himself choosing these instruments now in at least 75 per cent. of his operations.

DR. J. MCFADDEN GASTON, of Atlanta, read a paper entitled "Extirpation of the Rectum for Carcinoma."

The patient, a white man, aged sixty-two years, was referred to Dr. Gaston by Dr. J. I. Darby, of Columbia, Alabama. There was no family history of malignant disease, but his appearance led to the conclusion that there might be a cancerous cachexy. Having had occasion to report two cases of papilloma of the rectum with carcinomatous degeneration, which terminated fatally without operation, he recently advised in a similar case extirpation of the entire mass. This was done without serious consequences on May 30, 1891, and promised a good result, but there was a redevelopment of the induration above with a fatal result.

The patient referred to was received at the Providence Infirmary with rectal trouble. Upon digital examination and exploration with the speculum, there was found to exist an induration and thickening of the submucous tissues extending from an inch within the anus up to the recto-colic junction. The mucous membrane presented a dark conjested appearance attended with some oozing of blood. There was a marked contriction of the upper portion of the indurated structure, but admitting of the passage of the point of the index finger by forcible upward pressure, giving the impression that the structure above was not involved in the disease. It was therefore determined after consultation with several colleagues, that they had a case of carcinoma of the rectum of a circumscribed nature, which warranted an operation, and extirpation was performed July 8, 1891. The patient did well up to the 16th of July, when a marked change came about rather unexpectedly during the day, ending in collapse. At 11 P. M. he died of septicæmia.

DR. J. W. HALLUM, of Carrollton, contributed a paper on "The Treatment of Hemorrhoids by Carbolic Acid Injections."

In most of the cases that apply for relief it is unnecessary to delay, but the speaker proceeds at once to make a radical cure of the pile tumor. He does this by mixing together pure carbolic acid one part and pure glycerine two parts, and enough morphine and tannic acid that ten drops of mixture will contain one quarter of a grain of each. Let this be well shaken and thoroughly dissolved before using it.

The position that he has found to be probably the best is the one in which a toad assumes when in a sitting posture. In this position the patient will almost invariably pass out the tumors by an effort at straining down. There is more or less burning for a few minutes consequent from the injection, after which in the course of five or six hours the tumor begins to throb and ache from the swelling and also the death of the pile itself.

Adjourned.

THIRD DAY—AFTERNOON SESSION.

The Association re-assembled at 2:30 P. M., and after transacting some miscellaneous business, proceeded to the installation of officers. The following were elected:

President, Dr. A. A. Smith, Hawkinsville; First Vice-President, Dr. Geo. J. Grimes, Columbus; Second Vice-President, Dr. R. H. Taylor, Griffin; Treasurer, Dr. E. C. Goodrich, Augusta; Secretary, Dr. D. H. Howell, Atlanta.

On motion the association adjourned to meet in Americus, third Wednesday in April, 1893.

